# Acute and Chronic Effects of Whole-Body Vibration Training on Oxidative Stress and Cellular Damage Markers in Young Healthy Women

**DOI:** 10.3390/ijms27020899

**Published:** 2026-01-16

**Authors:** Halina Gattner, Justyna Adamiak, Olga Czerwińska-Ledwig, Sylwia Mętel, Magdalena Kępińska-Szyszkowska, Anna Kurkiewicz-Piotrowska

**Affiliations:** 1 Faculty of Physiotherapy, University of Physical Culture in Krakow, Jana Pawła II Avenue 78, 31-571 Krakow, Poland; 2 Institute of Applied Sciences, Faculty of Physiotherapy, University of Physical Culture in Krakow, Jana Pawła II Avenue 78, 31-571 Krakow, Poland; 3 Department of Chemistry and Biochemistry, Faculty of Physiotherapy, University of Physical Culture in Krakow, Jana Pawła II Avenue 78, 31-571 Krakow, Poland

**Keywords:** WBVT, oxidative stress, 8-hydroxy-2′-deoxyguanosine, myoglobin, muscle damage, DNA damage, TOS, TAC, healthy young women

## Abstract

The acute (single-session) and chronic (12-week) effects of whole-body vibration training (WBVT) on oxidative stress, muscle damage, and deoxyribonucleic acid (DNA) damage were evaluated in inactive women (20.48 ± 1.72 years). Participants were assigned to vibration training (EVG, *n* = 17), traditional exercise (EXG, *n* = 12), or control groups (CON, *n* = 17). Blood was collected pre- and post- the first and last sessions for EVG and EXG and at baseline and after 12 weeks for the CON. A significant main effect of time was observed for total antioxidant capacity (TAC, *p* < 0.001), indicating long-term enhancement of the antioxidant barrier across all groups. Analysis of change scores (Δ) revealed that the 12-week intervention significantly dampened the acute post-exercise response for white blood cells (WBCs, *p* < 0.001), neutrophils (NEUTs, *p* = 0.010), and myoglobin (Mb, *p* = 0.004), confirming systemic adaptation in both training groups. A significant reduction in total oxidant status (TOS, *p* = 0.042) was also noted between the first and last sessions. Significant main effects of group were found for WBCs, NEUTs, 8-hydroxy-2′-deoxyguanosine (8-OHdG), Mb, body mass, and fat-free mass, reflecting persistent baseline differences; however, no significant group-by-time interactions were identified. In conclusion, while WBVT did not show superior effects, it is a safe modality, comparable to traditional exercise, for improving oxidative stress tolerance and muscle recovery.

## 1. Introduction

Oxidative stress is a state in which the balance between the production of free radicals, including reactive oxygen species (ROS), and the body’s ability to neutralize them is disturbed [1]. Physical exercise is a physiological factor that increases the concentration of these molecules in the body, in skeletal muscle cells and in other organs, due to intensified metabolic processes and greater oxygen consumption. Under homeostatic conditions, the body counteracts exercise-induced oxidative stress through an enzymatic antioxidant system comprising superoxide dismutase (SOD), catalase (CAT) and glutathione peroxidase (GPx), as well as a nonenzymatic antioxidant network that includes vitamins C, E and A, carotenoids, flavonoids and glutathione [2].

However, excessively intense, prolonged, and exhaustive physical exercise can lead to an overproduction of free radicals, which may damage cellular components—including proteins, lipids, and deoxyribonucleic acid (DNA)—thereby contributing to development of various disease, muscle injury, reduced immunity, and accelerated aging. Conversely, adaptation to a disturbed pro-oxidant/antioxidant balance through systematic, moderate exercise enhances the body’s antioxidant defenses, i.e., its ability to cope with oxidants [2,3]. Regular physical activity stimulates immune functions and exerts anti-inflammatory effects [4,5]. The body’s defensive capacity against free radicals is markedly more efficient in individuals who regularly engage in sport than in inactive persons, and exercise-induced oxidative stress is significantly lower in physically active individuals compared with those leading a sedentary lifestyle [6,7].

It is widely recognized that disordered mechanical vibrations to which the human body is exposed, particularly in occupational settings, can be harmful to health [8,9,10]. At the same time, the available literature indicates that controlled exposure to vibrations with strictly defined parameters can produce positive therapeutic effects [11,12,13]. Physical exercises combined with whole-body vibration (WBV) are increasingly used in medical rehabilitation, health-oriented training, and sport [14]. However, their additional benefits compared with traditional dynamic and resistance exercise remain equivocal and controversial, mainly due to heterogeneous study protocols including both the training methods applied and the platform parameters used (frequency, amplitude, and type of vibration) [15].

Whole-body vibration training (WBVT) involves performing dynamic or static exercises on a device that generates vibrations. It is based on neuromuscular stimulation by sinusoidal mechanical oscillations transmitted throughout the body of a person standing on a vibration platform. The vibratory stimulus induces micro-stretching of muscle fibers, which leads to reflex muscle contractions [11,14]. Documented effects include increases in muscle strength, improvements in balance and flexibility, and increases in bone mineral density and blood flow [16,17,18,19,20]. WBVT may also beneficially affect the endocrine and nervous systems [21,22].

The aim of this study was to evaluate the effects of short-term (acute) and long-term (chronic) WBVT on markers of oxidative stress and cellular damage, compared with the same training performed without the WBV stimulus. It was hypothesized that a 12-week WBVT program would increase total antioxidant capacity (TAC) and decrease total oxidant status (TOS), 8-hydroxy-2-deoxyguanosine (8-OHdG), and myoglobin (Mb) compared with the same exercise performed without vibration and with a control condition. Additionally, we hypothesized that acute WBVT sessions would induce transient leukocytosis and neutrophilia that would attenuate after training, indicating adaptation.

## 2. Results

### 2.1. Subjects’ Characteristics

No significant baseline differences were observed among the exercise vibration (EVG), exercise (EXG), and control (CON) groups regarding physical characteristics or daily nutrient intake (all *p* > 0.05) (Table 1). Analysis of body composition revealed a significant group effect for body mass (BM; *p* = 0.020) and fat-free mass (FFM; *p* = 0.015). Regarding the temporal dynamics, no significant effect of time or group-by-time interaction was found for these parameters; however, notable statistical trends for the interaction effect were observed for FFM (*p* = 0.063) (Table 2). Consequently, post hoc comparisons (with Bonferroni correction) were performed on the data averaged across both time points (I and III). These tests revealed that the EVG had significantly lower BM (mean difference: 7.35 kg; *p* = 0.021) and lower FFM (mean difference: 3.57 kg; *p* = 0.013) compared to the CON.

### 2.2. Short-Term Effects of Exercise

#### 2.2.1. Acute Differential Leukocyte (White Blood Cell) Count

In the analysis of white blood cell (WBCs) and neutrophil (NEUTs) counts, similar statistical patterns were observed: significant main effects of time (*p* = 0.016 and *p* = 0.034, respectively) and group (*p* = 0.011 and *p* = 0.010, respectively) were found. The significant main effect of group indicates that, regardless of the time point, the EXG consistently exhibited higher values for both parameters compared to the EVG (Table 3). Due to the absence of a significant group-by-time interaction (noting a statistical trend for WBCs, *p* = 0.061), temporal dynamics were assessed based on the pooled data from both groups. Post hoc tests revealed:

- significant post-exercise increase (acute response): Both WBCs and NEUTs count showed a rapid elevation immediately following the first training session (WBCs: mean increase of 0.95 × 10^3^/µL, *p* < 0.001; for NEUTs 0.72 × 10^3^/µL, *p* = 0.002). A similar, though significantly attenuated, acute response was observed following the final session (III vs. IV), where NEUTs showed a statistically significant increase (*p* < 0.001; mean increase 0.24 × 10^3^/µL).
- effective recovery: Subsequent measurement points showed a significant decline in values. For WBCs, significant reductions relative to the post-exercise peak (II) were observed at point III (*p* = 0.025; mean decrease of 1.03 × 10^3^/µL) and point IV (*p* = 0.048; mean decrease of 0.90 × 10^3^/µL). For NEUTs, a borderline significant decrease was observed between points II and III (*p* = 0.05). These changes represent a return to levels comparable to the baseline (I) for both parameters (I vs. IV, *p* > 0.05), indicating a full return to physiological homeostasis.

An additional analysis of change scores (Δ II–I vs. Δ IV–III) confirmed a significant training-induced adaptation in the acute response. A significant main effect of time was found for both WBCs (*p* < 0.001), and NEUTs (*p* = 0.010), indicating a marked attenuation of post-exercise leukocytosis after the training period. No significant group-by-time interaction was observed (*p* > 0.05), suggesting that both training programs were similarly effective in reducing the acute inflammatory response to exercise (Table 4).

#### 2.2.2. Acute Oxidative Stress and Cellular Damage Indices

A statistically significant main effect of time was found for TOS (*p* = 0.003) (Table 5). Due to the lack of a significant group-by-time interaction, post hoc tests were performed on the data averaged across groups. These tests showed a significant decrease in the parameter level by 8.05 U/mL (*p* = 0.042) only between the measurement after the first training session (II) and after the last training session (IV). Furthermore, a non-significant numerical trend toward a decrease was observed between the baseline (I) and the final measurement (IV) (*p* = 0.074), suggesting a possible late-phase reduction in oxidative stress.

In the analysis of TAC, a significant main effect of time was found (*p* = 0.015). Additionally, a notable trend toward significance was observed for the main effect of group (*p* = 0.066), suggesting a tendency for the EVG to maintain higher TAC levels than the EXG across all time points (Table 5). Due to the absence of significant interaction, temporal dynamics were assessed based on the pooled results of both groups. Although the post hoc analysis did not reveal statistically significant differences between specific time points, a clear upward numerical trend was observed. Specifically, TAC levels showed a progressive increase relative to baseline (I), reaching a maximum mean elevation of 9.36 µmol/L at point IV (*p* = 0.081).

For the DNA damage marker—8-OHdG, significant main effects of time (*p* = 0.012) and group (*p* < 0.001) were observed. The significant effect of group indicates that the EXG consistently exhibited markedly higher 8-OHdG concentrations throughout the study compared to the EVG (Table 5). Although the overall ANOVA model showed a significant effect of time, post hoc analysis performed on the pooled data from both groups did not reveal statistically significant differences between specific time points (*p* > 0.05).

ANOVA showed significant main effects of time (*p* = 0.001) and group (*p* = 0.018) for Mb. The group effect was driven by systematically higher Mb concentrations in the EXG compared to the EVG across all time points (Table 5). Due to the lack of interaction, post hoc tests were performed on averaged data for the effect of time. A significant decrease of 6.34 ng/mL (*p* = 0.006) was observed specifically between the post-first (II) and post-last training (IV) measurements.

An additional ANOVA for change scores revealed a significant main effect of time only for Mb (*p* = 0.004), with no significant group-by-time interaction (*p* > 0.05). This indicates that both training programs were equally effective in inducing physiological adaptation, characterized by a significant attenuation of the acute post-exercise Mb release (Table 4).

### 2.3. The Effects of a 12-Week Training Program

#### 2.3.1. Chronic Differential Leukocyte (White Blood Cell) Count

A significant main effect of group was found for WBC levels (*p* = 0.019), with no significant effect of time or group-by-time interaction (Table 6). Consequently, post hoc comparisons were performed on the averaged data from both time points (I and III). These tests revealed that the EXG exhibited significantly higher WBCs compared to the EVG (*p* = 0.017), with an average difference of approximately 1.4 × 10^3^/µL maintained throughout the study.

#### 2.3.2. Chronic Oxidative Stress and Cellular Damage Indices

Long-term analysis (I vs. III) revealed a notable trend toward significance for the main effect of time regarding TOS (*p* = 0.060), suggesting a tendency for this parameter to decrease across all participants after the 12-week study (Table 7).

For TAC, only a significant main effect of time was observed (*p* < 0.001). After 12 weeks, a significant mean increase in TAC levels of 11.24 µmol/L was recorded across all participants. There was no significant main effect of group and no significant group-by-time interaction, indicating that the increase in TAC followed a similar pattern in all groups, including the control group (mean increases: EVG: 13.23; EXG: 4.8; CON: 15.7 µmol/L) (Table 7).

A significant main effect of group was found for 8-OHdG levels (*p* < 0.001) (Table 7). Post hoc tests performed on the data averaged across all time points revealed that the EVG had significantly lower concentrations compared to both the CON (mean difference: 1.48 ng/mL; *p* < 0.001) and the EXG (mean difference: 1.57 ng/mL; *p* < 0.001).

ANOVA revealed a significant main effect of group (*p* = 0.033) for Mb concentration (Table 7). Due to the absence of a significant group-by-time interaction, post hoc tests were performed on data averaged across all time points. These tests confirmed that the EVG had significantly lower Mb levels compared to the EXG (mean difference of 4.19 ng/mL; *p* = 0.029) throughout the 12-week study.

## 3. Discussion

To the best of our knowledge, this is the first study to assess the impact of WBVT on cellular damage markers: 8-OHdG and Mb in young, healthy, physically inactive women.

The primary finding of this study is that a 12-week training program led to significant systemic adaptation, characterized by a marked attenuation of the acute exercise-induced response for WBCs, NEUTs, and Mb. These adaptations were further accompanied by a significant reduction in absolute post-exercise TOS levels and a long-term increase in TAC. Importantly, the inclusion of WBV in the exercise protocol did not provide additional benefits, as both training programs were equally effective in inducing these physiological changes while maintaining the stability of long-term cellular health markers such as 8-OHdG. Furthermore, the observed increase in TAC across all groups, including the control group, suggests that factors beyond the specific training protocols, such as lifestyle adjustments during the study period, likely contributed to the enhanced antioxidant capacity.

The metabolic processes that activate during physical activity lead to increased production of free radicals, and their overproduction triggers oxidative stress [2]. During contractile activity, there is greater oxygen utilization by skeletal muscles. An increase in mitochondrial respiration enhances muscle metabolism, which in turn elevates electron flux through the respiratory chain and promotes the formation of free radicals [23,24,25]. Although ROS can damage lipids, proteins, and DNA, their production during exercise also serves as a signal that initiates and regulates adaptive changes in skeletal muscle, the myocardium, and internal organs such as the brain and liver [26]. The hormetic concept in the context of physical activity implies that both sedentariness and exercise that overloads the organism favor disease development, whereas regular, moderate training helps prevent it [27,28]. Disruption of internal homeostasis during exercise stimulates endogenous regulatory mechanisms at the molecular level. As oxidant production rises with exercise, the body responds with increased production and activity of antioxidants; with training, the organism adapts to an increased ROS production and more effectively counteracts their adverse consequences [2,28]. The body’s antioxidant capacity, including TAC, can be influenced mainly by age, sex, training status, and diet [29,30,31,32]. Therefore, we applied strict group-homogeneity criteria: participants were homogeneous with respect to sex (all women), age, and habitual physical activity. Because the principal enzymatic antioxidants such as SOD, CAT, and GPx are intracellular, plasma TAC primarily reflects low–molecular-weight, non-enzymatic antioxidants (e.g., urate, ascorbate, tocopherols, carotenoids, thiol-containing proteins) and therefore does not represent the full capacity of the body’s antioxidant defense. Some of these are exogenous antioxidants supplied with the diet [33,34]. To minimize dietary influences on TAC, participants were instructed not to change their eating habits during the experiment and to refrain from vitamin or mineral supplementation. In addition, a pre-study dietary assessment using food diaries enabled the inclusion of women with similar daily energy intake and comparable consumption of individual nutrients. Despite these strict dietary controls, a significant increase in TAC was observed across all groups, including the control, over the 12-week period. This phenomenon suggests that in a population characterized by low baseline physical activity, the transition to a structured study routine may have triggered systemic homeostatic optimization. Since dietary antioxidant intake remained stable and supplementation was excluded, this enhancement of the antioxidant barrier likely reflects a collective physiological response to lifestyle stabilization or seasonal environmental shifts. Furthermore, the absence of hormonal contraception and smoking among participants provided a stable physiological background, allowing these subtle, non-exercise-related improvements in systemic antioxidant capacity to manifest independently of the specific training intervention.

Physical exercise is a non-pharmacological strategy for preventing many diseases, in which antioxidant defense plays a key role [35]. However, according to the World Health Organization (WHO), 31% of adults and 80% of adolescents do not achieve the recommended level of physical activity and based on available data the proportion of adults and youth not meeting the global recommendation of at least 150 min of moderate-intensity activity per week is projected to reach 35% by 2030 relative to the 2010 baseline [36]. This situation is driven in part by contemporary lifestyles and technological advances, with sedentary work and screen time predominating [37,38,39]. Given the brief duration of individual sessions (approx. 15–20 min), WBVT appears to be an accessible form of physical activity or a complement to conventional training. It may be a particularly practical option for physically inactive individuals when initiating training, as it offers a manageable time commitment. Vibration platforms are widely available in fitness clubs, which are popular venues for promoting physical activity.

The number of studies examining the short- and long-term relationships between vibration training and redox status is limited. Moreover, comparing findings across vibration-therapy studies is challenging due to the wide range of vibration parameters employed in training protocols. Our results indicate a twofold response of the redox system to the 12-week intervention. Specifically, the training program successfully reduced exercise-induced oxidative stress, as evidenced by a significant decrease in TOS levels between the first and last sessions (II vs. IV). This suggests an effective systemic adaptation to chronic exercise stimuli. In contrast, the significant long-term increase in TAC levels observed across all groups, including the control indicates a cumulative effect of lifestyle stabilization and standardized dietary monitoring rather than a modality-specific response. The lack of a significant group-by-time interaction further suggests that while the exercise stimulus, potentially too subtle to elicit a rapid antioxidant surge, helped mitigate acute damage (TOS), the overall enhancement of the antioxidant barrier was a collective result of the 12-week study conditions. In a study by Wadsworth and Lark [40] comparing a single session and an 8-week program across three groups of women (WBV, downhill running, and treadmill walking), GPx activity increased after all exercise modalities, but to the smallest extent in the vibration group, suggesting lower ROS production. By contrast, changes in TAC were observed only after the first session in the downhill running and treadmill walking groups. The oxidative-stress marker F2-isoprostane (F2-IsoP) changed the least following vibration training. Theodorou et al. [41] assessed redox status by measuring thiobarbituric acid–reactive substances (TBARSs) and TAC in middle-aged women with low training status and found no effect of either a single WBV session or a repeated 8-week WBV program on oxidative-stress markers. Similarly, in young, physically active men a single WBV session did not alter TAS, TOS, or the oxidative stress index (OSI) [42]. In a cohort of 21 women, WBVT effects on oxidative-stress markers were compared between those with fibromyalgia (FM) and healthy controls (CG). A single WBVT session improved the pro-/antioxidant balance by increasing SOD and CAT activity in CG; in FM, it decreased TBARS and ferric-reducing antioxidant power (FRAP), lowered CAT, and increased SOD activity, findings consistent with a more favorable adaptation to oxidative stress in this group [43].

Systematic, moderate exercise exerts beneficial effects on the immune system and has an anti-inflammatory impact, including reduced secretion of acute-phase proteins and increased levels of circulating anti-inflammatory cytokines [5,44]. By contrast, overly intense exercise can cause micro-injuries to muscle tissue, leading to the release of intracellular proteins such as Mb, creatine kinase (CK), lactate dehydrogenase (LDH), and aspartate aminotransferase (AST) and to elevated C-reactive protein (CRP)—which is produced by the liver in response to inflammatory signals from the damaged muscle tissue. Most resistance exercises involve both concentric and eccentric muscle actions, with exercise-induced muscle damage occurring primarily during eccentric work [45]. Vibration training induces continuous, alternating eccentric–concentric muscle activity with increased oxygen consumption [46]. Depending on the duration and intensity of exercise, different mechanisms of innate (nonspecific) immunity are engaged. Post-exercise leukocytosis is a physiological response to physical stress, reflecting mobilization of leukocytes from tissue reservoirs into the bloodstream, and typically resolves within a few hours (within 24 h). Acute, vigorous effort initially elicits marked neutrophilia that persists for several hours after exercise. In addition, monocytosis and lymphocytosis are observed, along with an initial rise in natural killer (NK) cells that returns to resting values a few hours post-exercise. In the early recovery phase (from about 1 h after cessation of exercise), a transient immunosuppression may occur, characterized by decreases in lymphocytes (lymphocytopenia) and NK cells, together with an increase in NEUTs. This so-called “open-window” period may increase susceptibility to infection [45,47,48].

In our study, both the first and the last training sessions elicited significant increases in WBC and NEUT counts, with a more pronounced response observed at the beginning of the study. This acute elevation (point II) results from physiological exercise-induced leukocytosis, associated with cell demarginalization and an early response to exercise-induced muscle micro-injury. The attenuation of this reaction after 12 weeks, confirmed by the statistical comparison of pre- and post-intervention change scores, indicates systemic adaptation and the so-called repeated bout effect (RBE), suggesting increased muscle resistance to damage [49]. The absence of significant group-by-time interaction indicates that this process occurred similarly in both training groups. However, a significant main effect of group was observed for WBC (I–IV: *p* = 0.011; I–III: *p* = 0.019) and NEUT (I–IV: *p* = 0.010) levels, with the EXG consistently maintaining higher values than the EVG. This difference, likely resulting from a lack of full randomization and inter-individual variations, had no pathological basis. All results remained within clinical norms, confirming that the higher levels in the EXG represented their natural physiological homeostatic set-point rather than an inflammatory state.

Beyond damage to muscle cell membranes, intense and/or prolonged exercise can also induce adverse changes in other structures, including cellular DNA [50]. Because the respiratory chain resides in mitochondria, mitochondrial DNA is particularly vulnerable to ROS originating from electron leak in mitochondrial transport chain and prone to mutation. Mitochondria participate in aerobic respiration and adenosine triphosphate (ATP) production: during oxidative phosphorylation, electron-transport chain activity oxidizes nucleotides such as nicotinamide adenine dinucleotide (NADH) and flavin adenine dinucleotide (FADH_2_), generating energy that is subsequently used to synthesize ATP. This process is associated with free-radical production; thus, mitochondria constitute a major intracellular source of ROS [51,52]. Our findings regarding muscle and cellular damage markers suggest a twofold response to the 12-week intervention. The significant reduction in acute Mb response, supported by the comparison of acute change scores from the first and last training sessions, indicates a successful adaptive process. This is further evidenced by the decrease in absolute Mb levels between the first and last training sessions (II vs. IV), likely attributed to RBE, which enhances muscular resilience to mechanical stress. Conversely, the long-term stability of both Mb and 8-OHdG levels between baseline and the 12-week follow-up (I vs. III) indicates that the training protocols did not induce chronic muscle injury or cumulative DNA damage. It is also worth noting that 8-OHdG is a highly stable marker; its lack of change, coupled with the simultaneous increase in TAC, suggests that the repair mechanisms functioned efficiently, preventing the accumulation of damage. Notably, the significant main effects of group for both parameters reveal that the EXG consistently maintained higher concentrations of Mb and 8-OHdG compared to the EVG throughout the study. These persistent disparities likely stem from baseline inter-group differences rather than the intervention itself. The higher levels of damage markers in the EXG may be attributed to individual physiological characteristics or a higher initial susceptibility to oxidative stress among these participants. We found no studies assessing changes in myoglobin or 8-OHdG in response to WBVT, making it difficult to directly compare our results with those of other authors. Existing reports focus mainly on CK and LDH. Gojanovic et al. [53] reported that, in young physically inactive individuals, an intense WBVT protocol (32 Hz, 15 mm amplitude; total duration 27 min) can elicit a release of CK and LDH into the bloodstream. In another study using a 6 min WBV bout at 30 Hz and 4 mm amplitude, increases in rectus femoris stiffness and elevated CK persisted for 1 h post-exercise [54]. By contrast, in healthy, untrained adult women, neither a single session nor an 8-week WBVT program (20–25 Hz, 6 mm amplitude) altered muscle-damage markers (CK, LDH, AST) or CRP levels, leading the authors to conclude that the method is a safe form of training that does not induce adverse muscular effects [41]. Similarly, Wadsworth and Lark observed no change in CK in healthy young women after an 8-week vibration-platform training program performed three times per week (26 Hz, 6.8 mm amplitude) [40].

Several researchers suggest that applying either local vibration (50 Hz/1 min or 60 Hz/5 min) or WBV (35 Hz, 5 mm, 1 min) before eccentric exercise may attenuate exercise-induced muscle damage, manifested by lower CK levels and reduced delayed-onset muscle soreness (DOMS) [55,56,57]. Beneficial effects of WBV have also been observed after eccentric efforts: in a group additionally exposed to vibration at 12 Hz, 4 mm in three 1 min bouts, CK concentrations at 24 and 48 h were lower than in a control group performing eccentric exercise alone [58]. Similarly, Piotrowska et al. reported that local cycloidal–oscillatory vibrations reduced CK, LDH, and Mb compared with controls, both immediately and 24 h after a 180 min moderate-intensity cycling bout in young, physically active men [59].

### Limitations and Strengths

This study has several limitations. The main ones include unequal group sizes and the absence of randomization. Non-randomized group allocation may have introduced selection bias and baseline imbalances; thus, causal inference is limited and results should be interpreted as hypothesis-generating. Another limitation is the lack of additional blood sampling 24 h after sessions, which could have provided a more complete picture of the prolonged effects of WBVT on evaluated biochemical markers.

The study also has notable strengths. It involved three groups, allowing a more comprehensive analysis of the outcomes. Baseline assessments of diet and habitual physical activity enabled the selection of a homogeneous sample which is essential to obtain comparable results, especially in small groups. Moreover, carefully scheduled blood draws to measure the outcomes in the same phase of the menstrual cycle helped minimize the influence of fluctuations in sex hormone levels.

## 4. Materials and Methods

### 4.1. Participants

**Figure 1:**
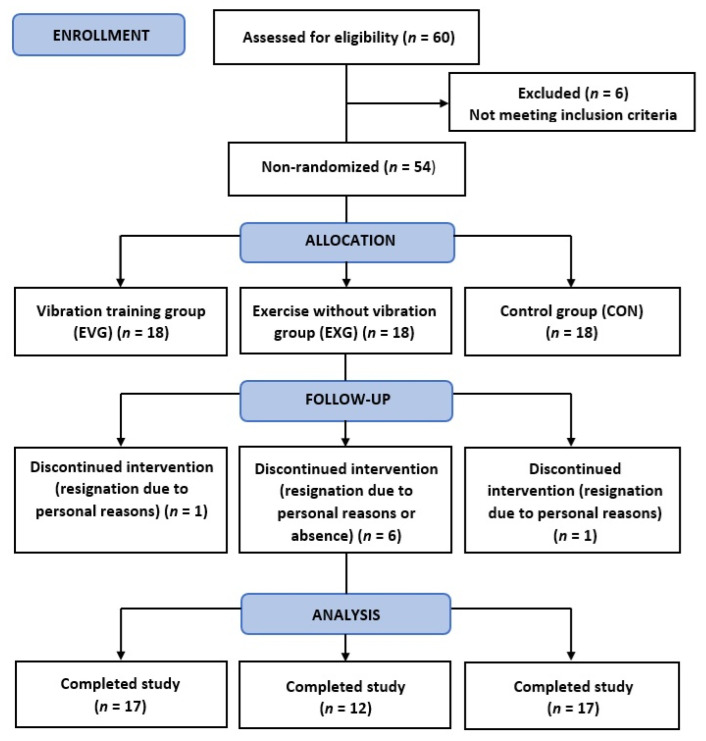
Study flow diagram.

The study sample initially comprised 54 women (mean age 20.48 ± 1.72 years), students of the University of Physical Education in Krakow recruited via a campus advertisement. Volunteers were allocated to one of three groups: a whole-body vibration training group (EVG, *n* = 17 included in the final analysis), an exercise group performing the same exercises without vibration (EXG, *n* = 12 included in the final analysis), and a control group with no intervention (CON, *n* = 17 included in the final analysis). Age, height, baseline total energy intake, and the main dietary components are presented in Table 1. Table 2 summarizes body-composition characteristics. The participant flow diagram is shown in Figure 1.

Inclusion criteria were female sex, age 18–25 years, low physical activity level assessed using the short version of the International Physical Activity Questionnaire (IPAQ) [60]; normal weight range as a body mass index (BMI) 18.5–24.9 kg/m^2^, according to WHO recommendations [61]; a diet consistent with national guidelines [62]; and no contraindications to WBVT confirmed by medical screening [63]. Exclusion criteria comprised smoking, hormonal contraception, polycystic ovary syndrome or anovulatory cycles, special/elimination diets in the previous 3 months, and regular use of antioxidant vitamins or other supplements during the month before baseline. The homogeneous profile was chosen to reduce biological and lifestyle variance in redox outcomes. Participants were fully informed about the study protocol and their right to withdraw at any time without consequences. The study was approved by the Bioethics Committee of the Regional Medical Chamber in Krakow (224/KBL/OIL/2016) and conducted in accordance with the Declaration of Helsinki. The project was registered in a clinical-trials registry (trial ID: ACTRN12621000114842).

### 4.2. Study Protocol

Before allocation to a specific group, each participant underwent: a medical interview and examination, assessment of physical activity and diet, and body-composition analysis. Recruitment and selection for each of the three groups were conducted separately from the pool of volunteers. Participants assigned to the 12-week WBVT group and to the exercise-only group underwent venous blood sampling four times: immediately before and after the first training session and immediately before and after the last session. In the CON, blood was collected twice within a 3-month interval. All participants underwent repeat body-composition analysis after 12 weeks.

### 4.3. Body Composition Analysis

In all women, BM was measured before and after the study using a Tanita BC-418 MA analyzer (measurement error: 0.1 kg; Tanita, Tokyo, Japan), and body composition was estimated by bioelectrical impedance analysis (BIA). For each participant, the following variables were assessed: BMI (kg/m^2^); percent body fat (PF, %); fat mass (FM, kg); FFM (kg); and total body water (TBW, kg). Body height was measured using a stadiometer.

### 4.4. Nutritional Analysis and Physical Activity Level

Before the experiment, diet was assessed using 5-day food diaries (4 weekdays and 1 weekend day) and analyzed with Dieta 5 software (Institute of Food and Nutrition, Warsaw, Poland) [62]. Baseline total energy intake, macronutrients, and vitamins A, C, and E did not differ between groups (Table 1). Participants were instructed to maintain their habitual diet and to avoid vitamin/mineral supplements throughout the study. Habitual physical activity was screened with the short form of IPAQ [60]. Participants had low habitual physical activity at baseline and were asked not to change their lifestyle during the intervention. Day-to-day dietary variation and unmeasured lifestyle changes cannot be fully excluded, which we acknowledge as a limitation.

### 4.5. Venous Blood Collection and Biochemical Assays

Venous blood was drawn from the antecubital vein in the morning, after an overnight fast, with participants in the seated position, by a certified laboratory diagnostician. A BD Vacutainer vacuum system was used (Becton Dickinson, Franklin Lakes, NJ, USA). Blood was collected into two tube types: dipotassium ethylenediaminetetraacetic acid (K_2_EDTA) tubes for leukocyte indices and clot-activator tubes for oxidative-stress, muscle-damage, and DNA-damage markers. In EVG and EXG, venous blood was collected four times: immediately before and after the first training session and before and after the last session. In CON, blood was collected twice at a 3-month interval.

WBCs and differentials: NEUTs, monocytes (MONOs), eosinophils (EOs), and basophils (BASOs); units × 10^3^/μL were determined in an external accredited medical diagnostics laboratory by flow cytometry using a Sysmex XN-9000 analyzer (Sysmex Corporation, Kobe, Japan).

Clot-activator tubes were centrifuged at 3500 rpm for 10 min at 4 °C (MPW-350R, MPW Med. Instruments, Warsaw, Poland). Serum was aliquoted into Eppendorf-type microtubes and stored at −80 °C in a low-temperature freezer (ULF 390, Arctiko Dairei, Esbjerg, Denmark) until analysis.

Serum TAC (μmol/L), TOS (U/mL = μmol H_2_O_2_ equivalents/L), 8-OHdG (ng/mL), and Mb (ng/mL) were measured by enzyme-linked immunosorbent assay (ELISA) using high-sensitivity kits (DRG Instruments GmbH, Marburg, Germany; SunRed Bio, Shanghai, China). Absorbance was read at 450 nm on an ELISA microplate reader (ChroMate 4300, Awareness Technology Inc., Palm City, FL, USA). Assay sensitivities were: TAC 130 μmol/L; TOS 1.362 U/mL; 8-OHdG < 0.938 ng/mL; Mb 5 ng/mL. Intra-assay and inter-assay coefficients of variation were as follows: TAC: intra 3.0%, inter 3.3%; TOS: intra < 8%, inter < 10%; 8-OHdG: intra < 8%, inter < 10%; Mb: intra 5.5%, inter 8.3%.

### 4.6. Exercise Program

**Figure 2:**
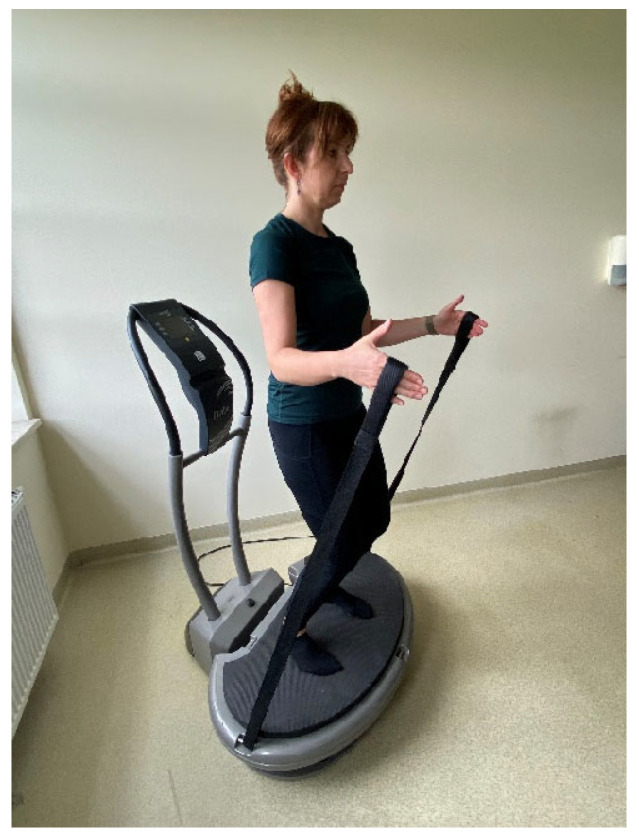
Back-to-platform standing position while holding tensioned straps.

**Figure 3:**
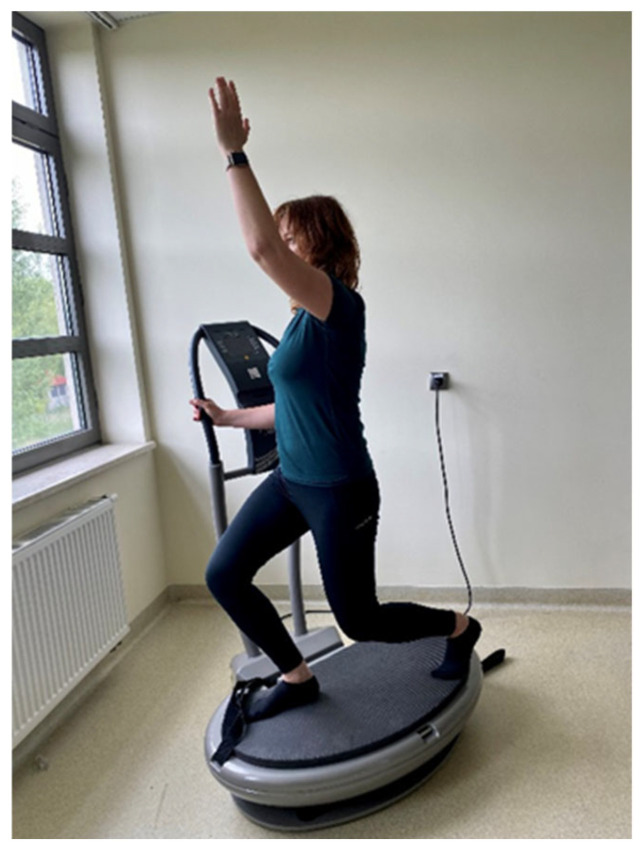
Dynamic squat in a forward lunge on the platform.

WBVT was delivered individually on a linear (vertical) platform (Fitvibe Excel Pro, Gymna Uniphy, Bilzen, Belgium) at 2 mm amplitude, with frequency progression of 40 Hz (sessions 1–12), 45 Hz (13–24), and 50 Hz (25–36). Each session comprised six 1 min work bouts (three static tasks: standing; back-to-platform with strap tension; half-squat hold; and three dynamic tasks: half-squats and side-squats in a forward lunge to the right and left lower limb with a simultaneous lateral raise of the contralateral upper limb) (Figure 2 and Figure 3), paced at 25 repetitions·min^−1^ for dynamic tasks, and separated by 1 min walking rests (work:rest 1:1). Main-part exposure on the platform was 6 min per session (plus 5 min off-platform rest between bouts), with a 3 min warm-up (three revitalizing/stretching exercises performed standing, 6 repetitions each) and a 3 min cool-down (three relaxing floor-based exercises on a mat, 6 repetitions each). The total duration of a single training session was approximately 20 min. The exercise-only group performed the same sequence without vibration under supervision, in small groups of 3–4 participants (the first and last sessions were conducted individually). Training sessions in both groups were performed three times per week for 12 weeks. The control group had no intervention. The total WBV exposure was 216 min over 36 sessions. A detailed description of the specific exercises has been published previously [20]. No adverse events or injuries related to the exercise or vibration protocols were reported.

### 4.7. Statistical Analysis

All statistical analyses were performed in IBM SPSS Statistics, version 24 (IBM Corp., Armonk, NY, USA). Data are presented as mean ± standard deviation (SD). Normality was assessed with the Shapiro–Wilk test. An a priori power analysis was performed in G*Power 3.1.9.7 (Heinrich Heine University, Düsseldorf, Germany) for a F test (repeated-measures ANOVA, within–between interaction) with three groups and four measurement points. Assuming a medium effect size (Cohen’s f = 0.25), based on conventional recommendations in the absence of prior empirical estimates, α = 0.05, power (1 − β) = 0.85, an assumed correlation among repeated measures of r = 0.50, and a nonsphericity correction ε = 1.0, the required total sample size was approximately *n* = 36 (12 participants per group). We enrolled 54 participants; 46 completed the protocol and were analyzed.

Depending on data distribution, baseline between-group differences in physical characteristics and nutrition were examined using one-way ANOVA or the Kruskal–Wallis test. To evaluate the effects of time and group, as well as their interaction, a two-way mixed ANOVA was employed. Post hoc analyses were conducted only when statistically justified by significant main effects or interactions. In the case of short-term effects, where the group-by-time interaction was not significant, significant main effects of time were followed by pairwise comparisons using Bonferroni-adjusted post hoc tests on pooled data (collapsed across the EVG and EXG). For long-term effects, when the group × time interaction was absent, but a significant main effect of group was identified, Bonferroni-adjusted post hoc tests were performed to determine specific differences between the three groups (EVG, EXG, and CON) regardless of the time point. Furthermore, to evaluate the influence of the training programs on the magnitude of the acute exercise response, a 2 × 2 mixed ANOVA was performed on the change scores, comparing the pre-training acute response (Δ II–I) with the post-training acute response (Δ IV–III) between groups. Statistical significance was set at α = 0.05. The effect size was assessed using the Eta squared coefficient (η^2^) and interpreted as follows: 0.1—small; 0.25—medium; 0.37—large size effect [64].

## 5. Conclusions

The 12-week training program effectively induces beneficial systemic adaptation, characterized by dampened post-exercise inflammatory and muscular damage responses, confirming the occurrence of the ‘repeated bout effect.’ Both training protocols were equally effective, indicating that the inclusion of WBV does not provide additional benefits. This training is a safe modality that improves overall antioxidant capacity and reduces absolute post-exercise oxidative stress without inducing chronic inflammation or DNA damage, while the observed increase in TAC highlights the crucial role of lifestyle stabilization in optimizing redox homeostasis.

### Practical Application

Given the health benefits of regular physical activity and the fact that a substantial proportion of young people do not meet recommended activity levels, there is a continued need to explore and understand the mechanisms of action of alternative forms of exercise, including WBVT. Due to the brief nature of the protocol (approx. 15–20 min per session), platform-based exercises may be particularly useful in the initial phase of training among physically inactive individuals or as an attractive complement to conventional training. However, as no additional benefits of WBVT were observed over traditional exercise in this study, these findings require confirmation in future studies utilizing a full randomization protocol to further evaluate its potential role in the prevention of lifestyle-related diseases closely linked to ROS production.

## Figures and Tables

**Table 1:** Age, body height and diet analysis results (performed at baseline).

Parameter	EVG (*n* = 17)	EXG (*n* = 12)	CON (*n* = 17)	*p*
Age [years]	21.65 ± 1.8	20.17 ± 1.75	19.53 ± 0.72	0.064
Body height [cm]	162.76 ± 7.51	164.67 ± 5.94	167.24 ± 4.56	0.187
Energy [kcal]	1713.26 ± 651.45	1688.97 ± 326.07	1683.32 ± 602.52	0.915
Protein [g]	65.41 ± 18.15	65.15 ± 18.06	66.49 ± 22.92	0.991
Fat [g]	54.23 ± 24.73	53.15 ± 14.64	50.26 ± 21.32	0.926
Carbohydrates [g]	220.73 ± 54.98	231.04 ± 47.43	240.01 ± 75.86	0.856
Vitamin A [µg]	812.59 ± 333.40	650.51 ± 306.94	708.53 ± 226.39	0.417
Vitamin E [mg]	6.30 ± 2.24	5,99 ± 1.52	5.65 ± 1.86	0.657
Vitamin C [mg]	72.86 ± 35.95	59.59 ± 29.39	64.35 ± 37.59	0.486

Values are mean ± standard deviation; exercise vibration group (EVG); exercise group (EXG); control group (CON).

**Table 2:** Body composition measurements.

		Baseline (I)	After 12 Weeks (III)	Δ III–I	ANOVA *p* Value (η^2^)		
					Time	Group	G × T
BM [kg]	EVG	56.57 ± 7.18	56.64 ± 6.72	0.07 ± 1.72	0.217 (0.036)	0.020 (0.163)	0.384 (0.045)
	EXG	59.43 ± 6.04	59.48 ± 5.62	0.05 ± 1.24			
	CON	63.62 ± 8.89	64.29 ± 8.88	0.67 ± 1.06			
BMI [kg/m^2^]	EVG	21.31 ± 1.87	21.54 ± 1.93	0.22 ± 0.61	0.355 (0.020)	0.320 (0.053)	0.434 (0.039)
	EXG	22.02 ± 2.91	21.98 ± 2.68	−0.04 ± 0.5			
	CON	22.65 ± 2.5	22.69 ± 2.38	0.12 ± 0.52			
PBF [%]	EVG	23.04 ± 6.11	24.01 ± 5.65	0.97 ± 3.51	0.690 (0.004)	0.278 (0.059)	0.091 (0.108)
	EXG	25.62 ± 4.14	24.27 ± 5.39	−1.35 ± 2.14			
	CON	26.59 ± 5.77	26.48 ± 5.31	0.22 ± 2.12			
FM [kg]	EVG	13.34 ± 4.54	13.86 ± 4.23	0.52 ± 2.1	0.826 (0.001)	0.086 (0.110)	0.152 (0.086)
	EXG	15.4 ± 3.85	14.65 ± 4.46	−0.75 ± 1.34			
	CON	17.34 ± 5.93	17.41 ± 5.5	0.07 ± 1.43			
FFM [kg]	EVG	43.24 ± 3.81	42.78 ± 3.57	−0.45 ± 1.81	0.132 (0.053)	0.015 (0.181)	0.063 (0.116)
	EXG	44.03 ± 2.84	44.83 ± 2.74	0.8 ± 1.26			
	CON	46.26 ± 3.35	46.9 ± 3.91	0.64 ± 0.97			
TBW [kg]	EVG	31.65 ± 2.79	31.32 ± 2.62	−0.33 ± 1.32	0.120 (0.057)	0.065 (0.111)	0.063 (0.110)
	EXG	32.22 ± 2.07	32.81 ± 2.01	0.59 ± 0.89			
	CON	33.87 ± 2.46	34.34 ± 2.87	0.47 ± 0.7			

Values are mean ± standard deviation; η^2^ = eta squared; G × T: group-by-time interaction; body mass (BM); body mass index (BMI); percentage body fat (PBF); fat mass (FM); fat free mass (FFM); total body water (TBW); exercise vibration group (EVG); exercise group (EXG); control group (CON).

**Table 3:** Changes in differential leukocyte (white blood cell) count after a single bout of exercise.

Parameter		I	II	Δ II–I	III	IV	Δ IV–III	Δ IV–II	ANOVA *p* Values (η^2^)		
									Time	Group	G × T
WBC [10^3^/µL]	EVG	5.01 ± 0.98	5.87 ± 0.86	0.86 ± 0.76	5.21 ± 0.85	5.57 ± 1.02	0.36 ± 0.5	−0.30 ± 0.45	0.016 (0.171)	0.011 (0.219)	0.061 (0.122)
	EXG	6.68 ± 2.7	7.71 ± 2.7	1.03 ± 1.1	6.31 ± 1.45	6.23 ± 1.63	−0.08 ± 0.48	−1.48 ± 1.35			
NEUT [10^3^/µL]	EVG	2.64 ± 0.78	3.34 ± 0.79	0.7 ± 0.69	2.83 ± 0.78	3.19 ± 0.86	0.36 ± 0.27	−0.15 ± 0.77	0.034(0.140)	0.010(0.219)	0.124(0.081)
	EXG	4.27 ± 2.46	5.01 ± 2.67	0.74 ± 1.19	3.53 ± 1.18	3.63 ± 1.27	0.10 ± 0.26	−1.38 ± 2.65			
LYMPH [10^3^/µL]	EVG	1.86 ± 0.32	1.86 ± 0.45	0 ± 0.33	1.81 ± 0.31	1.81 ± 0.38	0 ± 0.25	−0.06 ± 0.21	0.397 (0.034)	0.299 (0.040)	0.118 (0.075)
	EXG	1.99 ± 0.64	1.86 ± 0.46	−0.13 ± 0.46	2.12 ± 0.42	1.98 ± 0.48	−0.14 ± 0.24	0.12 ± 0.23			
MONO [10^3^/µL]	EVG	0.42 ± 0.11	0.46 ± 0.11	0.04 ± 0.09	0.44 ± 0.08	0.43 ± 0.07	−0.01 ± 0.08	−0.03 ± 0.11	0.631 (0.021)	0.066 (0.120)	0.287 (0.045)
	EXG	0.52 ± 0.16	0.49 ± 0.12	−0.03 ± 0.13	0.53 ± 0.14	0.48 ± 0.14	−0.05 ± 0.08	−0.01 ± 0.09			
EO[10^3^/µL]	EVG	0.11 ± 0.06	0.1 ± 0.07	−0.01 ± 0.03	0.12 ± 0.07	0.11 ± 0.08	0 ± 0.02	0.01 ± 0.07	0.451 (0.026)	0.834 (0.02)	0.559 (0.017)
	EXG	0.11 ± 0.07	0.1 ± 0.06	−0.01 ± 0.02	0.11 ± 0.04	0.1 ± 0.04	−0.01 ± 0.01	0 ± 0.04			
BASO [10^3^/µL]	EVG	0.03 ± 0.01	0.03 ± 0.02	0 ± 0.01	0.03 ± 0.01	0.03 ± 0.01	0 ± 0.01	0 ± 0.02	0.755 (0.015)	0.062 (0.121)	0.824 (0.011)
	EXG	0.04 ± 0.01	0.04 ± 0.02	0 ± 0.011	0.04 ± 0.02	0.04 ± 0.02	0 ± 0.01	0 ± 0.01			

Values are mean ± standard deviation; η^2^ = eta squared; G × T: group-by-time interaction; exercise vibration group (EVG); exercise group (EXG); white blood count (WBC); neutrophil (NEUT); lymphocyte (LYMPH); monocyte (MONO); eosinophil (EO); basophil (BASO); I—measurement performed before the first exercise training (baseline); II—measurement performed after the first exercise training; III—measurement performed before the last exercise training; IV –measurement performed after the last exercise training.

**Table 4:** Statistical analysis of changes in the acute effect of exercise (comparison of change scores: Δ II–I vs. Δ IV–III).

Parameter	ANOVA *p* Value (η^2^)		
	Time	Group	G × T
WBC [10^3^/µL]	<0.001 (0.432)	0.537 (0.014)	0.100 (0.097)
NEUT [10^3^/µL]	0.010 (0.222)	0.575 (0.012)	0.400 (0.026)
LYMPH [10^3^/µL]	0.920 (3.761 × 10^−4^)	0.184 (0.064)	0.986 (1.118 × 10^−5^)
MONO [10^3^/µL]	0.165 (0.070)	0.068 (0.118)	0.502 (0.017)
EO [10^3^/µL]	0.481 (0.019)	0.452 (0.021)	0.862 (0.001)
BASO [10^3^/µL]	0.710 (0.005)	0.852 (0.001)	0.361 (0.031)
TOS [U/mL]	0.205 (0.059)	0.338 (0.034)	0.480 (0.019)
TAC [µmol/L]	0.161 (0.071)	0.633 (0.009)	0.200 (0.060)
8-OHdG [ng/mL]	0.104 (0.095)	0.298 (0.040)	0.877 (9.095 × 10^−4^)
Mb [ng/mL]	0.004 (0.273)	0.522 (0.015)	0.143 (0.078)

η^2^ = eta squared; G × T: group-by-time interaction; white blood count (WBC); neutrophil (NEUT); lymphocyte (LYMPH); monocyte (MONO); eosinophil (EO); basophil (BASO); total oxidant status (TOS) [U/mL] = [μmol H_2_O_2_ Eqv/L]; total antioxidant capacity (TAC); 8-hydroxy-2′-deoxyguanosine (8-OHdG); myoglobin (Mb).

**Table 5:** Changes in oxidative stress and cellular damage indices after single bout of exercise.

Parameter		I	II	Δ II–I	III	IV	Δ IV–III	Δ IV–II	ANOVA *p* Values (η^2^)		
									Time	Group	G × T
TOS [U/mL]	EVG	53.19 ± 46.49	54.77 ± 46.19	1.58 ± 5.41	50.38 ± 41.82	46.07 ± 37.2	−4.31 ± 9.76	−8.70 ± 13.4	0.003 (0.173)	0.173 (0.076)	0.729 (0.014)
	EXG	34.72 ± 22.07	36.32 ± 24.85	1.60 ± 10.77	28.73 ± 19.97	28.92 ± 21.31	0.2 ± 9.03	−7.4 ± 14.37			
TAC [µmol/L]	EVG	369.71 ± 19.28	378.94 ± 14.3	9.23 ± 25.35	382.94 ± 12.12	379.13 ± 16.01	−3.8 ± 14.86	0.19 ± 11.75	0.015 (0.124)	0.066 (0.110)	0.512 (0.029)
	EXG	332.83 ± 6.69	337.9 ± 12.4	5.07 ± 10.36	337.63 ± 8.81	342.13 ± 9.35	4.50 ± 11.44	4.23 ± 7.82			
8-OHdG [ng/mL]	EVG	3.19 ± 0.67	3.13 ± 0.32	−0.06 ± 0.8	3 ± 0.56	2.72 ± 0.46	−0.28 ± 0.64	−0.41 ± 0.37	0.012 (0.132)	<0.001 (0.751)	0.267 (0.056)
	EXG	4.51 ± 1.22	4.84 ± 1.3	0.33 ± 1.19	4.81 ± 0.49	4.79 ± 0.99	−0.02 ± 0.89	−0.05 ± 0.99			
Mb[ng/mL]	EVG	13.69 ± 5.26	16.37 ± 7.22	2.68 ± 8.16	12.94 ± 5.37	12.15 ± 5.16	−0.79 ± 7.04	−4.22 ± 8.38	0.001 (0.197)	0.018 (0.198)	0.445 (0.033)
	EXG	17.17 ± 5.45	21.52 ± 8.61	4.35 ± 5.67	17.84 ± 5.23	13.05 ± 4.65	−4.79 ± 6.35	−8.47 ± 9.8			

Values are mean ± standard deviation; η^2^ = eta squared; G × T: group-by-time interaction; total oxidant status (TOS) [U/mL] = [μmol H_2_O_2_ Eqv/L]; total antioxidant capacity (TAC); 8-hydroxy-2′-deoxyguanosine (8-OHdG); myoglobin (Mb); exercise vibration group (EVG); exercise group (EXG); I—measurement performed before the first exercise training (baseline); II—measurement performed after the first exercise training; III—measurement performed before the last exercise training; IV—measurement performed after the last exercise training.

**Table 6:** Changes in differential leukocyte (white blood cell) count after 12-week training program.

Parameter		I	III	Δ III–I	ANOVA *p* Values (η^2^)		
					Time	Group	G × T
WBC[10^3^/µL]	EVG	5.01 ± 0.98	5.21 ± 0.85	0.2 ± 0.91	0.944(0.000)	0.019(0.169)	0.648(0.020)
	EXG	6.68 ± 2.7	6.31 ± 1.45	−0.37 ± 2.81			
	CON	5.47 ± 1.52	5.58 ± 1.36	0.11 ± 1.24			
NEUT[10^3^/µL]	EVG	2.64 ± 0.78	2.83 ± 0.78	0.19 ± 0.81	0.382(0.018)	0.326(0.045)	0.313(0.053)
	EXG	4.27 ± 2.46	3.53 ± 1.18	−0.74 ± 2.72			
	CON	3.15 ± 1.31	3.05 ± 1.11	−0.10 ± 1.13			
LYMPH[10^3^/µL]	EVG	1.86 ± 0.32	1.81 ± 0.31	−0.05 ± 0.29	0.077(0.071)	0.121(0.093)	0.093(0.105)
	EXG	1.99 ± 0.64	2.12 ± 0.42	0.13 ± 0.45			
	CON	1.68 ± 0.29	1.89 ± 0.4	0.21 ± 0.35			
MONO[10^3^/µL]	EVG	0.42 ± 0.11	0.44 ± 0.08	0.02 ± 0.11	0.233(0.033)	0.054(0.127)	0.857(0.007)
	EXG	0.52 ± 0.16	0.53 ± 0.14	0.01 ± 0.11			
	CON	0.44 ± 0.09	0.47 ± 0.11	0.03 ± 0.1			
EO[10^3^/µL]	EVG	0.11 ± 0.06	0.12 ± 0.07	0.01 ± 0.05	0.634(0.005)	0.527(0.029)	0.703(0.016)
	EXG	0.11 ± 0.07	0.11 ± 0.04	0 ± 0.05			
	CON	0.17 ± 0.32	0.13 ± 0.06	−0.04 ± 0.31			
BASO[10^3^/µL]	EVG	0.03 ± 0.01	0.03 ± 0.01	0 ± 0.01	0.856(0.001)	0.166(0.080)	0.899(0.005)
	EXG	0.04 ± 0.01	0.04 ± 0.02	0 ± 0.01			
	CON	0.04 ± 0.02	0.04 ± 0.02	0 ± 0.02			

Values are mean ± standard deviation; η^2^ = eta squared; G × T: group-by-time interaction; exercise vibration group (EVG); exercise group (EXG); control group (CON); white blood count (WBC); neutrophil (NEUT); lymphocyte (LYMPH); monocyte (MONO); eosinophil (EO); basophil (BASO); I—measurement performed in rest on the first day of training program (baseline); III—measurement performed in rest on the last day of training program.

**Table 7:** Changes in oxidative stress and cellular damage indices after 12-week training program.

Parameter		I	III	Δ III–I	ANOVA *p* Values (η^2^)		
					Time	Group	G × T
TOS[U/mL]	EVG	53.19 ± 46.49	50.38 ± 41.82	−2.81 ± 15.07	0.060(0.124)	0.480(0.040)	0.703(0.019)
	EXG	34.72 ± 22.07	28.73 ± 19.97	−5.99 ± 10.4			
	CON	53.77 ± 67.22	46.13 ± 55.62	−7.64 ± 18.55			
TAC[µmol/L]	EVG	369.71 ± 19.28	382.94 ± 12.12	13.23 ± 22.18	<0.001(0.410)	0.131(0.071)	0.608(0.023)
	EXG	332.83 ± 6.69	337.63 ± 8.81	4.8 ± 12.35			
	CON	352.36 ± 42.38	368.06 ± 29.52	15.7 ± 41.59			
8-OHdG[ng/mL]	EVG	3.19 ± 0.67	3 ± 0.56	−0.19 ± 0.87	0.956(0.000)	<0.001(0.476)	0.514(0.034)
	EXG	4.51 ± 1.22	4.81 ± 0.49	0.3 ± 1.11			
	CON	4.61 ± 1.26	4.53 ± 1.14	−0.08 ± 1.1			
Mb[ng/mL]	EVG	13.69 ± 5.26	12.94 ± 5.37	−0.75 ± 6.72	0.686(0.004)	0.033(0.160)	0.757(0.014)
	EXG	17.17 ± 5.45	17.84 ± 5.23	0.67 ± 7.55			
	CON	15.37 ± 5.55	14.22 ± 4.03	−1.15 ± 4.98			

Values are mean ± standard deviation; η^2^ = eta squared; G × T: group-by-time interaction; total oxidant status (TOS) [U/mL] = [μmol H_2_O_2_ Eqv/L]; total antioxidant capacity (TAC); 8-hydroxy-2′-deoxyguanosine (8-OHdG); myoglobin (Mb); exercise vibration group (EVG); exercise group (EXG); control group (CON); I—measurement performed in rest on the first day of training program (baseline); III—measurement performed in rest on the last day of training program.

## Data Availability

The original contributions presented in this study are included in the article. Further inquiries can be directed to the corresponding author.
